# Modified Scoring of the QuickDASH Can Achieve Previously-unattained Interval-level Measurement in Dupuytren Disease and Carpal Tunnel Syndrome

**DOI:** 10.1097/GOX.0000000000005372

**Published:** 2024-02-08

**Authors:** Paul H.C. Stirling, Jane E. McEachan, Jeremy N. Rodrigues, Luke Geoghegan, Conrad J. Harrison

**Affiliations:** From the * Queen Margaret Hospital, Dunfermline, Scotland, UK; † Fife Virtual Hand Clinic, Dunfermline, Scotland, UK; ‡ Department of Plastic Surgery, Stoke Mandeville Hospital, Buckinghamshire Healthcare NHS Trust, UK; § Warwick Clinical Trials Unit, Warwick Medical School, University of Warwick, Coventry, UK; ¶ Section of Vascular Surgery, Department of Surgery and Cancer, Imperial College London, London, UK; ‖ Nuffield Department of Orthopaedics, Rheumatology and Musculoskeletal Sciences, University of Oxford, Oxford, UK.

## Abstract

**Background:**

Rasch measurement theory can be used to identify scales within questionnaires and to map responses to more precise continuous scales. The aim of this article was to use RMT to refine the scoring of the QuickDASH in patients with Dupuytren disease and carpal tunnel syndrome (CTS).

**Methods:**

Data were collected between 2013 and 2019 from a single center in the UK. Preoperative QuickDASH responses from patients diagnosed with Dupuytren disease and CTS were used. RMT was used to reduce the number of items in the QuickDASH and examine the reliability and validity of each subscale.

**Results:**

The preoperative QuickDASH responses of 750 patients with Dupuytren disease and 1916 patients with CTS were used. The median age of participants was 61 years, and 46% were men. Exploratory factor analysis suggested two distinct subscales within the QuickDASH: task items 1–6 and symptom items 9–11. These items were fitted to the Rasch model, and disordered response thresholds were collapsed. In Dupuytren disease, the two worst responses or each item were disordered. After collapsing these options, good Rasch model fit was demonstrated. CTS responses fitted without modification. Item targeting was more appropriate for CTS than Dupuytren disease.

**Conclusions:**

This study proposes a modification to the scoring system for the QuickDASH that provides high-quality, continuous, and condition-specific scales for the QuickDASH. The identification of distinct subscales within the QuickDASH can be used to identify distinct improvements in hand function and/or symptoms in previous, current, and future work.

**Takeaways**

**Question:** Can the QuickDASH provide true continuous measurement of function and symptoms using Rasch measurement theory?

**Findings:** Preoperative QuickDASH responses from patients with carpal tunnel syndrome (n = 1916) and Dupuytren disease (n = 750) were used. Exploratory factor analysis suggested two distinct subscales (function items 1–6 and symptoms items 9–11). These items were fitted to the Rasch model, and good fit was demonstrated.

**Meaning:** This study proposes a modification to the scoring system of the QuickDASH. The identification of distinct subscales with interval-level scoring can be used to identify improvements in hand function and/or symptoms that may not have been detected through composite scoring.

## INTRODUCTION

The Disabilities of the Arm, Shoulder, and Hand (DASH) questionnaire is the most used patient-reported outcome measure in hand surgery research.^1^ The DASH comprises 30 items designed to measure upper extremity function and symptoms scored on a five-point Likert scale.^2^ To reduce response burden, a shortened version of the DASH was derived in 2005.^3^ The QuickDASH comprises 11 items scored on a five-point Likert scale and has comparable psychometric properties to the full-length PROM.^4^ Both the DASH and the QuickDASH are scored on a scale from 0 to 100, with higher scores indicating greater disability. Item response scores are summed and averaged, producing a score out of five. This value is then scaled by subtracting one and multiplying by 25.^5^

The interpretation of individual patient scores can be challenging. Contemporary cross-sectional data suggest that patients with a score of less than 15 are generally asymptomatic, whereas those with a score of greater than 40 are unable to work due to their upper limb function and/or symptoms.^5^ Both the DASH and the QuickDASH assume that all items reflect a single underlying entity (are “unidimensional”) and involve fixed scoring of items using ordinal scales, which assumes that score intervals are always equally spaced. If unidimensionality is assumed inappropriately, and ordinal scores are treated as continuous data, clinically relevant changes may not be detected, or may be implied incorrectly.

Both issues may affect the QuickDASH. Firstly, previous work has indicated that different items that comprise the QuickDASH may measure different health constructs (or “factors”): for example, the degree of difficulty performing daily tasks or the severity of hand symptoms.^6,7^ Although symptoms and function may be related, they are conceptually different constructs. Summing symptom item scores and function item scores into a single overall score can make the interpretation of the QuickDASH problematic: positive change in one factor may be masked by negative change in another, for example.

Secondly, ordinal scales like QuickDASH are less accurate, precise, and interpretable than continuous scales because their measurement intervals are not equally spaced. For example, the difference in function between having mild and moderate problems holding a shopping bag is not necessarily equal to the difference in function between having moderate and severe problems holding a shopping bag. It cannot be assumed that the difference in function between a QuickDASH score of 5 and 10 is the same as the difference between a score of 10 and 15. Despite this, QuickDASH scores are often treated as continuous data in clinical practice and research, and so may fail to capture clinically relevant differences, or imply difference erroneously.

Rasch measurement theory (RMT) is a branch of psychometrics developed in the 1960s but only recently applied to surgical outcome measurement.^8^ It assumes unidimensionality and addresses the problem of ordinal scoring by mapping item responses to continuous scales through statistical models. In RMT, the probability of endorsing a given response is a function of the measured trait (eg, hand function). In other words, RMT can calculate the probability distribution of endorsing a certain response option, given the respondent’s level of hand function, or it can predict the respondent’s level of hand function on a continuous scale given a set of item responses.

Dupuytren contracture and carpal tunnel syndrome (CTS) are two of the most common conditions encountered in elective hand surgery^9^ and represent distinct symptom profiles. The aim of this study was to use RMT to determine whether the QuickDASH questionnaire could provide true continuous measurement in patients with these conditions.

### Methods

#### Patient Cohort

The study setting was a regional hand surgery center. A total of 750 patients who had surgical treatment for Dupuytren contracture and 1916 patients who underwent carpal tunnel decompression between 2013 and 2019 were identified. The median age was 61 (interquartile range: 51–71 years), and there were 1217 male patients (46%). Complete preoperative QuickDASH items responses were available for 731 patients (97%) with Dupuytren disease and 1851 patients with CTS (95%). Procedures were done under the care of a single consultant hand surgeon.

#### RMT Analysis

A previous exploratory factor analysis demonstrated unidimensionality of items 1–6 (“task-based items”) and items 9–11 (“symptoms-based items”).^7^ We undertook a separate RMT analysis for each group of items. The responses to these items are assessed on a five-point Likert scale (“no difficulty,” “mild difficulty,” “moderate difficulty,” “severe difficulty,” and “unable”).

Analyses were undertaken using *R*, version 4.0.3. First, the ability to consistently order items, a requirement for subsequently fitting to the Rasch model, was investigated using nonparametric item response theory-based Mokken analysis.^10^ In this, item scalability was assessed using Loevinger H coefficient, with a coefficient of greater than 0.3 considered acceptable.^10^ Local dependency (LD), where items are excessively related to each other, was measured by Yen’s Q3 statistic: values of more than 0.2 were accepted as indicating LD.^11^ Fit to the Rasch model assumes that LD is not present.

Item characteristic curves (ICCs) were plotted; item response thresholds were determined to investigate successive scoring of response options. The fit of each item to the Rasch model was assessed using a chi-square test, as well as infit and outfit mean squares. Chi-square tests that were nonsignificant at the level of *P* greater than 0.05 were deemed to represent appropriate model fit, along with infit and outfit mean squares between 0.5 and 1.7.

Close or disordered ICC thresholds were collapsed, and RMT analysis was repeated. Where item-level misfit still existed after response option collapse, we attempted to understand why misfit was occurring by using a generalized additive model^12^ to plot smoothed regression lines for item response probability functions in the mirt *R* package (version 1.33.2).^13^ Item-person plots were created to compare the targeting of items to the score distribution in our sample.

To assess scale-level fit, five model fit statistics were determined and reported: chi-square (χ^2^), comparative fit index (CFI), Tucker-Lewis index (TLI), root mean squared error of approximation (RMSEA), and the standardized root mean squared residual (SRMR). The following values were considered to indicate good Rasch model fit: χ^2^ *P* greater than 0.05, CFI greater than or equal to 0.950, TLI greater than or equal to 0.950, RMSEA less than 0.060, and SRMR less than or equal to 0.080.^14^ We anticipated type I errors in the item-level and scale-level χ^2^ *P* values, as these are almost always significant with sample sizes as large as ours.^15^ Internal consistency was assessed and reported as Cronbach alpha: a value of more than 0.7 represents acceptable internal consistency, while values 0.95 or more can suggest item redundancy.^16^

After RMT analysis, we calculated conversion tables to translate the “raw” ordinal sum score of the items to a continuous scale between 0 and 100. This was achieved by cross-walking matched sum scores and Rasch scores that were calculated via an expected a posteriori approach.^12^

### Results

#### RMT Analysis in Patients with Dupuytren Disease

Both groups of items demonstrated scalability with a Loevinger H coefficient of more than 0.3 (overall scale 0.733 for task-based; 0.721 for symptoms-based). No item pairs were locally dependent.

**Fig. 1.**
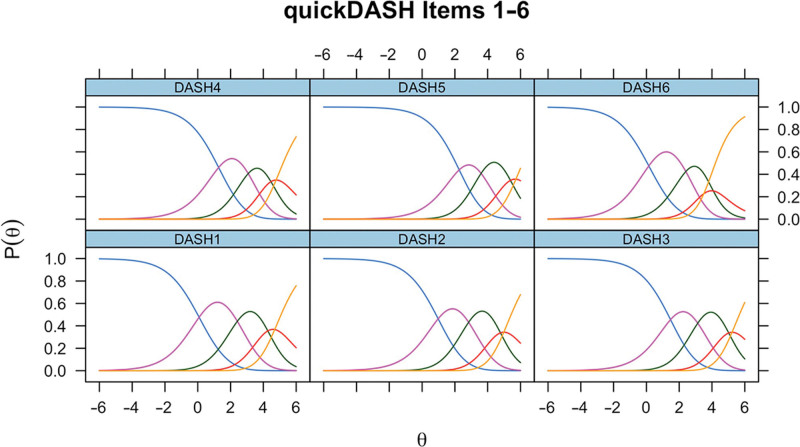
ICCs for unmodified QuickDASH items 1–6 in Dupuytren disease.

**Fig. 2.**
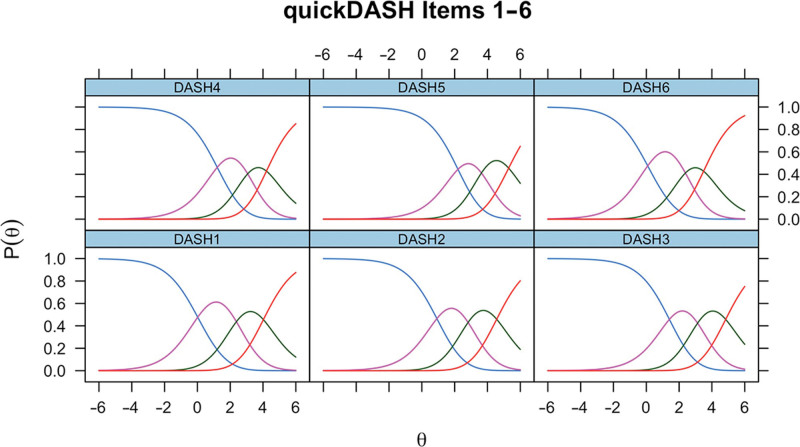
ICCs for items 1–6 in Dupuytren disease, following modification of the scoring system by collapsing response options 4 and 5.

**Fig. 3.**
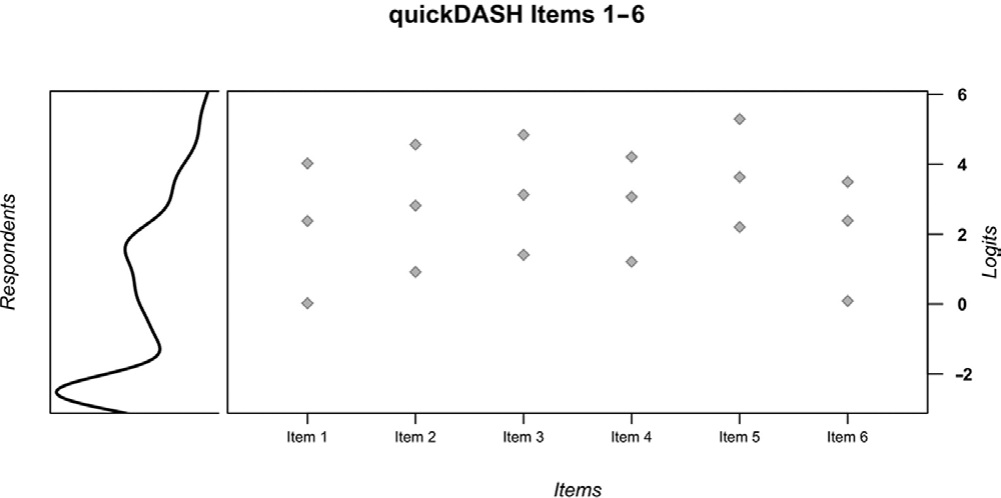
Item-person plots for the modified Rasch model for items 1–6 in CTS (top plot) and Dupuytren disease (bottom plot).

ICCs generated in the initial RMT assessment of QuickDASH items 1–6 in Dupuytren disease are presented in Figure 1. For all six items, the thresholds between the two most negative response options (“severe difficulty” and “unable”) were close or disordered in all items. What this means, in real-world terms, is that the difference between these two responses, in units of hand function, was either very small or nonexistent. We handled this by rescoring these response options equally. After rescoring the participant’s QuickDASH responses in this way, item-level fit was generally good, except for χ^2^ *P* values (Table 1). Items 2 (do heavy household chores), 3 (carry a shopping bag or briefcase), and 5 (use a knife to cut bread) showed poorer fit (outfit <0.5). Generalized additive model response curve estimates suggested that these items were poorly discriminative at the higher level of the scale even after collapsing the most severe response options; people generally did not have poor enough hand function to report severe difficulty in doing heavy chores, carrying shopping bags, or using a knife to cut food. (**See figure 1, Supplemental Digital Content 1,** which displays the generalized additive model plot for QuickDASH item 2 in Dupuytren disease. http://links.lww.com/PRSGO/C838.) (**See figure 2, Supplemental Digital Content 2,** which displays the generalized additive model plot for QuickDASH item 3 in Dupuytren disease. http://links.lww.com/PRSGO/C839.) (**See figure 3, Supplemental Digital Content 3,** which displays the generalized additive model plot for QuickDASH item 5 in Dupuytren disease. http://links.lww.com/PRSGO/C840.)

**Table 1.** Item-level Fit Statistics for Dupuytren Item Responses

Item	Question	Outfit	Infit	χ^2^ *P*
DASH1	Open a tight or new jar	0.695	0.922	<0.01
DASH2	Do heavy household chores	0.410	0.605	<0.01
DASH3	Carry a shopping bag or briefcase	0.333	0.549	<0.01
DASH4	Wash your back	0.709	0.984	<0.01
DASH5	Use a knife to cut bread	0.436	0.819	<0.01
DASH6	Recreational activities where you take some force through your hand	0.684	0.926	<0.01

Scale-level fit statistics were as follows: χ^2^ *P* less than 0.001, CFI = 0.980, TLI = 0.980, RMSEA = 0.097, and SRMR = 0.079. ICCs for the Dupuytren-adjusted scoring are demonstrated in Figure 2. Cronbach alpha was 0.924.

In our Dupuytren disease cohort, the IPP showed a negatively skewed score distribution, with items generally targeted toward discriminating between respondents with poorer hand function than were present in the sample (Fig. 3). Items 2, 3, and 5 were the most mis-targeted. The Rasch score conversions for items 1–6 are presented in Table 2.

**Table 2.** Conversion of Raw to Rasch Modified QuickDASH Scores for Items 1–6

CTS		Dupuytren Disease	
Raw QuickDASH Score	Rasch QuickDASH Score	Raw QuickDASH Score	Rasch QuickDASH Score
6	0	6	0
7	9	7	19
8	16	8	30
9	21	9	37
10	26	10	43
11	30	11	49
12	34	12	53
13	37	13	57
14	41	14	61
15	44	15	65
16	47	16	69
17	50	17	72
18	53	18	76
19	56	19	80
20	59	20	84
21	62	21	89
22	65	22	93
23	68	23	97
24	72	24	100
25	75		
26	79		
27	83		
28	88		
29	93		
30	100		

**Fig. 4.**
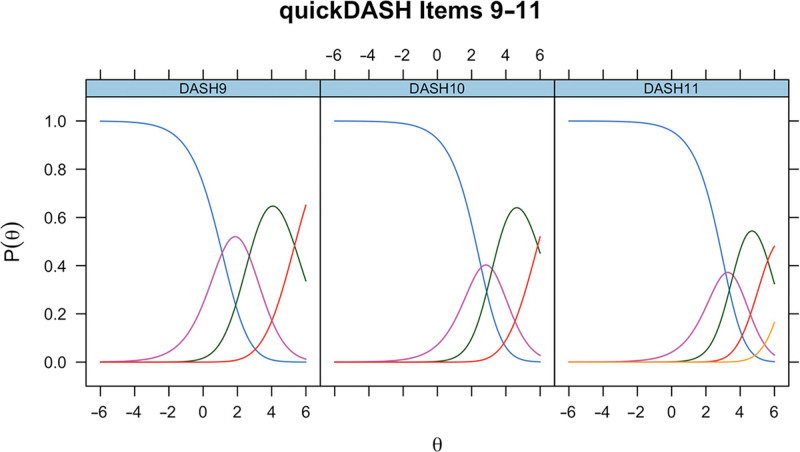
ICCs for unmodified QuickDASH items 9–11 in Dupuytren disease.

ICCs generated in the initial RMT assessment of QuickDASH items 9–11 (rate the severity of pain in the last week, rate the severity of tingling in the past week, and during the past week how much difficulty have you had sleeping) in Dupuytren disease are presented in Figure 4. For items 9–11, scale-level fit statistics were as follows: χ^2^ *P* less than 0.001, CFI = 0.980, TLI = 0.980, RMSEA = 0.086, and SRMR = 0.095. QuickDASH items 9–11 showed inappropriate targeting and a suboptimal Rasch model fit. As a result, no further modification was undertaken, although the Rasch score conversions for items 9–11 are presented in Table 3 for completeness.

**Table 3.** Conversion of Raw to Rasch Modified QuickDASH Scores for Items 9–11

Carpal Tunnel Syndrome		Dupuytren Disease	
Raw QuickDASH Score	Rasch QuickDASH Score	Raw QuickDASH Score	Rasch QuickDASH Score
3	0	3	0
4	15	4	27
5	27	5	43
6	38	6	54
7	48	7	63
8	59	8	71
9	71	9	79
10	85	10	88
11	100	11	95
		12	100

#### RMT Analysis in Patients with CTS

Loevinger H was more than 0.3 for items 1–6 (overall scale 0.704). There was no LD: all item pairs had a Yen Q3 of 0.2 or less.

**Fig. 5.**
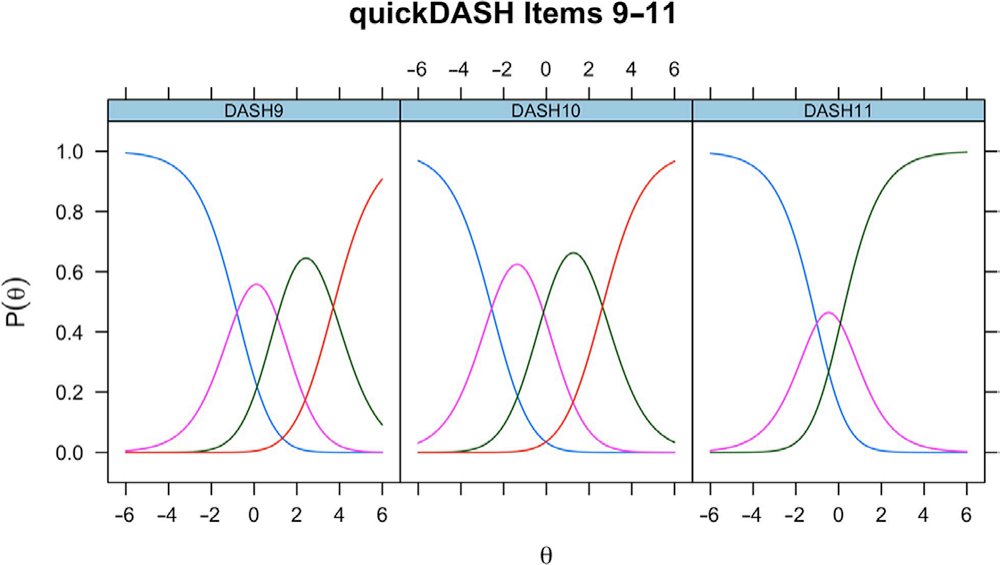
ICCs for the modified QuickDASH items in CTS.

ICCs from our CTS cohort are presented in Figure 5. No disordered thresholds were observed, and therefore, no modification was made to the scoring. Item-level fit statistics, except for χ^2^ *P* values, suggested good model fit for all items (Table 4). Scale-level fit statistics were as follows: χ^2^ *P* less than 0.001, CFI = 0.962, TLI = 0.962, RMSEA = 0.128, and SRMR = 0.062.

**Table 4.** Item-level Fit Statistics for CTS

Item	Outfit	Infit	χ^2^ *P*
DASH1	0.804	0.841	<0.01
DASH2	0.616	0.647	<0.01
DASH3	0.667	0.694	<0.01
DASH4	0.993	1.098	<0.01
DASH5	0.717	0.804	<0.01
DASH6	0.832	0.877	<0.01

The IPP demonstrated excellent targeting, with no floor or ceiling effects, and appropriately spaced thresholds (Fig. 3). The Rasch score conversions for items 1–6 are presented in Table 2; conversions for items 9–11 are presented in Table 3. To generate valid Dupuytren disease Rasch scores from QuickDASH items 1–6, clinicians and researchers should allocate scores of 4 to both the “severe difficulty” and “unable” response options. The item scores can then be summed and converted using our Rasch conversion tables. In CTS, no modifications are required at the item level: the scores from items 1–6 can be summed and converted with the table presented. For the symptoms-based items 9 and 10 in CTS, “no difficulty” should remain with a score of 1, “mild difficulty” should also be scored as 1, “moderate difficulty” should be scored as 2, “severe difficulty” should be scored as 3, and “unable” should be scored as 4. The same scoring system should be applied for item 11, but “unable” should also be scored as 3. Following this modified scoring, the conversion tables for items 9–11 can then be consulted. We do not recommend the use of items 9–11 in patients with Dupuytren disease. The Rasch scores are not interchangeable between Dupuytren disease and CTS, and the disease-specific conversion scores must be used.

**Fig. 6.**
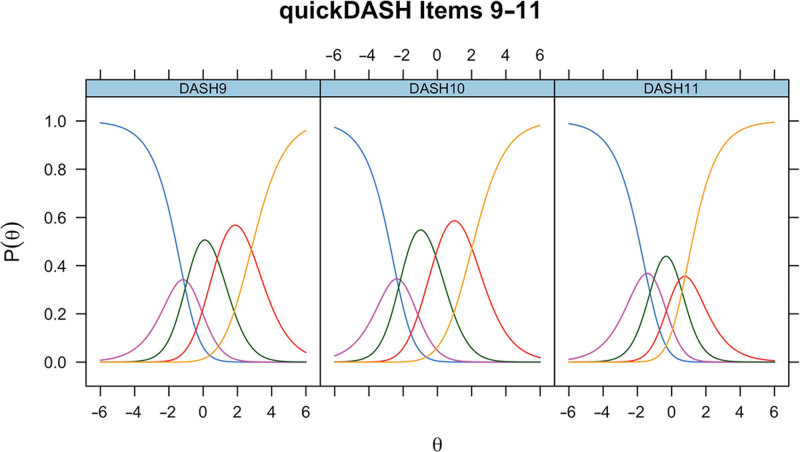
ICCs for unmodified QuickDASH items 9–11 in CTS.

**Fig. 7.**
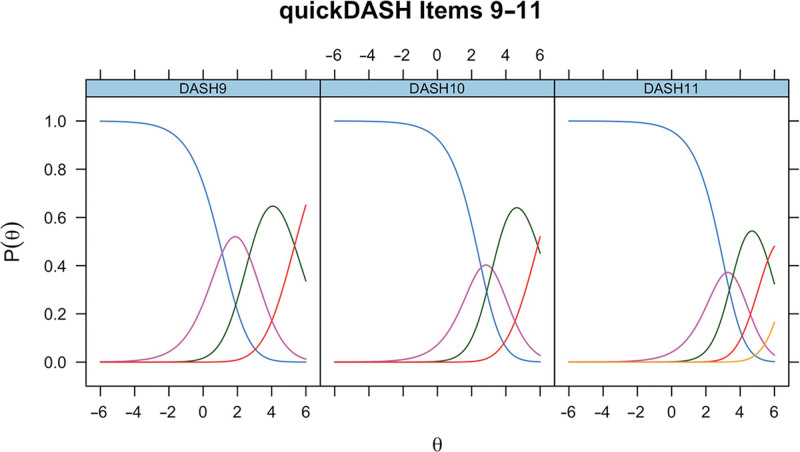
ICCs for the modified QuickDASH items 9–11 in Dupuytren disease.

**Fig. 8.**
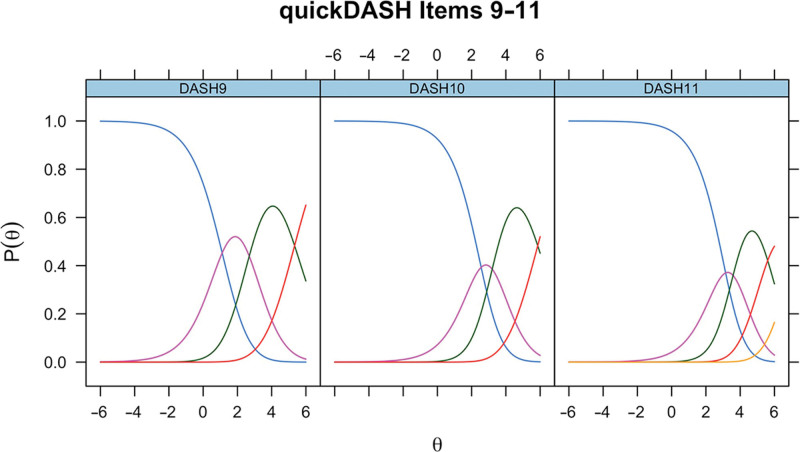
ICCs for unmodified QuickDASH items 9–11 in Dupuytren disease.

For completeness, the ICCs for unmodified QuickDASH items 9–11 in CTS for the modified QuickDASH items 9–11 in Dupuytren disease and for unmodified QuickDASH items 9–11 in Dupuytren disease are presented (Figs. 6–8).

### Discussion

We have previously suggested that QuickDASH items 1–6 can be used as a stand-alone, unidimensional measure of hand function in CTS and Dupuytren disease.^7^ This study has built on these results by providing a new condition-specific scoring system for these items, which allows accurate measurement of hand function on a continuous scale in patients with CTS and Dupuytren disease.

Rasch model scales detailed in the present study can be applied prospectively or retrospectively to QuickDASH response data to achieve more precise, accurate, and interpretable measurement of hand function in CTS and Dupuytren disease. This has immediate implications for clinicians at an individual patient level. For example, a patient presenting with CTS and a raw QuickDASH score of 8 (for items 1–6) has an equivalent Rasch QuickDASH score of 16. This clinically relevant, conventional scoring indicates the patient is likely asymptomatic, whereas Rasch scoring indicates the patient is likely to have problems working.

The primary strength of this study is that these modified scoring systems can be retrospectively applied to existing datasets. The increasing use of PROMs in different hand conditions has resulted in large datasets of generic PROM responses, although these instruments have not all been validated to contemporary psychometric standards. If large volumes of data have previously been collected, then it is desirable to use this for further study, if possible. Furthermore, modification of such datasets that leads to optimization of existing hand surgery PROMs has clear benefits, such as minimizing further inconvenience to patients, and streamlining research, while avoiding the significant costs which can be associated with the development, validation, and application of new PROMs. Such an approach could prove valuable while we await the development of new instruments that meet the criteria for validity and reliability set out in the consensus-based standards for the selection of health measurement instruments [Consensus-based Standards for the Selection of Health Measurement Instruments (COSMIN)] statement.^17^

There are also limitations to our study. Although this study could improve the structural validity of the QuickDASH in CTS and Dupuytren disease, we are unable to address the content validity of this PROM in these conditions. This represents the main limitation of this study. The QuickDASH is a short-form derivative of the DASH PROM^18^ developed through item reduction. The DASH was originally developed as a generic site-specific tool, before the introduction of the COSMIN checklist. Maintaining content validity is an important consideration when modifying pre-existing PROMs^19^: because the QuickDASH is not a condition-specific PROM, its validity may vary between conditions,^20^ with worse preoperative QuickDASH observed in patients with CTS than those with Dupuytren disease. There are a range of possible explanations for this observation.^21^ Patients presenting for treatment of Dupuytren disease may genuinely experience less disability than may be expected in other conditions affecting the hand. Indeed, previous work does indicate that a notable proportion undergo treatment to “prevent worsening” rather than to reverse impaired function per se.^22^ “Response shift,” where patients adapt to the slowly-progressing disability over time^23^ in Dupuytren disease, could also result in a lower level of self-reported disability in this patient cohort. These explanations are not mutually exclusive. In addition, questionnaire items that relate primarily to severity of numbness or tingling (item 10) are unlikely to be as relevant in patients with Dupuytren disease compared with patients with CTS. This could explain the variable targeting described in our study, and confirms the need to use specific Rasch scales for each condition. A further limitation of this study is that our sample was obtained from a single site. Potential differential item function by language or culture could reduce the usefulness of these scoring systems when applied in other countries or healthcare systems.

Overall, this study has generated continuous, condition-specific scales for a modified QuickDASH score, which could support notably improved measurement of function in CTS and Dupuytren disease. Findings from this study can be used immediately by clinicians to support shared decision-making in patients with CTS and Dupuytren disease. However, findings from this study cannot be generalized across all upper extremity conditions; future work should determine the generalizability of this work to other conditions and patient populations, and assess the real-world impact of this scoring modification. For example, studies could test whether repeating previous analyses with valid, unidimensional Rasch scoring changes a study’s outcome, improves the instrument’s responsiveness or test-retest reliability, or drives down sample size requirements for trials by reducing spurious population variance.

In summary, this study proposes a modification to the scoring system of the QuickDASH. The identification of distinct subscales with interval-level scoring can be used to identify improvements in hand function and/or symptoms that may not have been detected through composite scoring.

## DISCLOSURE

The authors have no financial interest to declare in relation to the content of this article.

## ACKNOWLEDGMENTS

Conrad J. Harrison is funded by a National Institute for Health Research (NIHR) Doctoral Research Fellowship (NIHR300684). Jeremy N. Rodrigues is funded by a NIHR postdoctoral fellowship (PDF-2017-10-075). This document presents independent research funded by the NIHR. The views expressed are those of the authors and not necessarily those of the NHS, the NIHR, or the Department of Health and Social Care.

## Supplementary Material


