# Supplementary figures and images for: Modified Scoring of the QuickDASH Can Achieve Previously-unattained Interval-level Measurement in Dupuytren Disease and Carpal Tunnel Syndrome
Source: Plast Reconstr Surg Glob Open. 2024 Feb 8;12(2):e5372. doi: 10.1097/GOX.0000000000005372 (PMC10852374; doi:10.1097/GOX.0000000000005372)

# GAM item probability curves

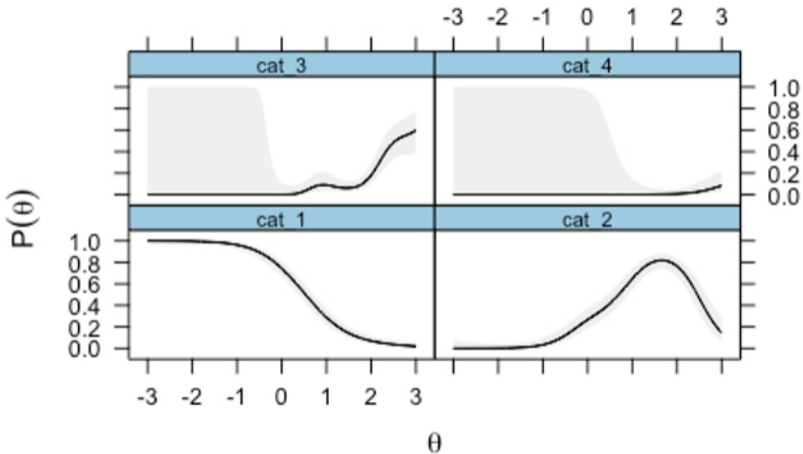

Supplement: Supplementary file 1 [file gox-12-e5372-s001.pdf]

# GAM item probability curves

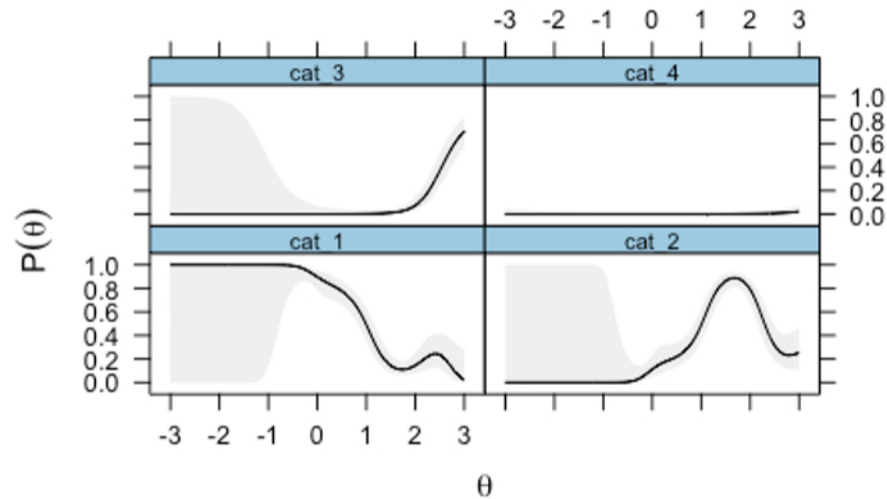

Supplement: Supplementary file 2 [file gox-12-e5372-s002.pdf]

# GAM item probability curves

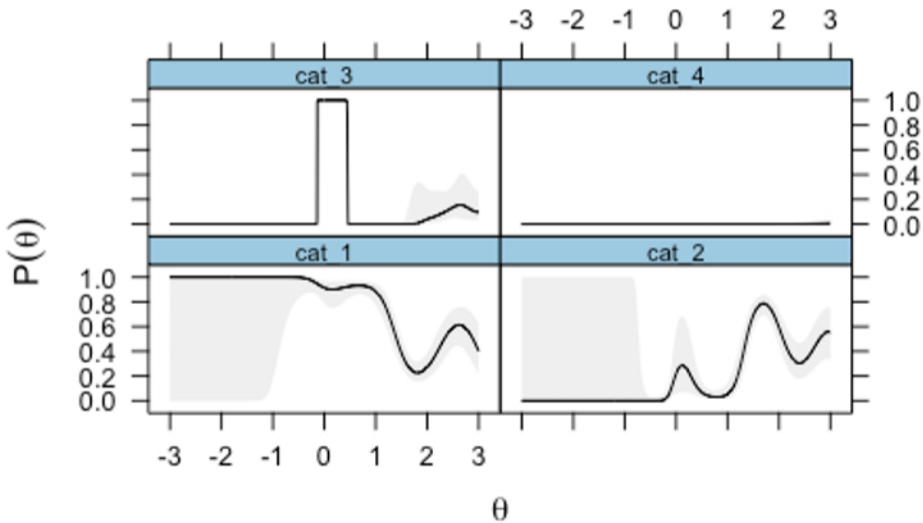

Supplement: Supplementary file 3 [file gox-12-e5372-s003.pdf]
